# Science and Art

**Published:** 1909-12-11

**Authors:** 


### Science and Art.

Apropos of a book of Mr. Masefield's, a medical contemporary has lately questioned the fundamental dissimilarity in the respective parts played by the imagination in these two great forms of human mental activity. True, they cannot rightly be opposed (as, for instance, Charles Kingsley opposed them) merely as analysis and synthesis, because most people are agreed that for the highest forms of scientific analysis some preliminary synthesis is required. The correct issue, however, is stated—after the fortunes of Mr. Masefield's hero, a dramatic author turned pathologist, have been followed for a little—by comparison of the eminence of Titian's portrait of the Emperor Charles V, with the eminence of the researches of Faraday: it being then argued that each is due to a quality of mind in artist and experimenter—a quality which we call imagination in Titian and insight in Faraday, but which might just as warrantably be styled Faraday's imagination and Titian's insight. As usual, there is force in what our contemporary says, but we think that further probing of the matter will show the unsoundness of his position. And it may as well be stated first as last, that there is a fundamental difference, namely this: the great artist's imagination is constructive—that of the great scientist, reconstructive.

To adopt the illustration given, large numbers in the sixteenth century could picture to themselves the great personality of the opponent of Luther and conqueror of Barbarossa; some perhaps could appreciate it better than Titian was able to. But he alone had constructive power to render it on canvas as it should be rendered, to make adequate visible representation of it. Examples of the same thing can be found in the history of all forms of art. Keats must have had cultured and sensitive contemporaries who felt emotion at the sight of a Greek vase. He alone was poet, or, to use the old English synonym, "maker," enough to body forth such emotion in a perfect ode. On the other hand, Faraday, at the date of his first great discovery, alone in his generation knew the principle of the induction coil; nobody else, from consideration of seemingly disconnected phenomena, had mentally reconstructed the physical law concerned. But anyone made acquainted with this principle could construct the visible representation of it with a hand's turn or two. The two forms of imaginative activity are, in truth, as far apart as generalisation from natural phenomena and creation of artificial objects. Again, we might take two present-day descriptions of art by writers as distinguished, and as modern in spirit, as Mr. Masefield. It is remoulding the world nearer one's heart's desire says one, and the other—whatever acts by exciting emotion. The worth of these characterisations we do not pretend to judge, but clearly neither of them affords any encouragement to the notion that Faraday's imagination acted like Titian's. The scientist does not construct a new little world of his own; he finds out how the old one is governed. His appeal is not at all to emotion, but solely to intellect. Jumping to right conclusions is the scientific use of the imagination, or rather the use of the scientific imagination: how the artist uses his is not for us to say, but even in architecture, the most scientific of the arts, it cannot be thus.

The subject is one which may legitimately be touched in a medical journal, because misconception and mistrust as to the application of the full scientific method to medical investigation probably still does harm. It is usual to hear the words "working with preconceived ideas" used opprobriously, and truths revealed by experiment quoted in confirmation; as though any great experiment were not inspired by a plan embodying the prevision of its result. The work of men with the power of original induction is regarded as elevating and interesting indeed, but of no practical significance. They are allowed to be rather notable enthusiasts—poets in their way, in fact—but are not taken seriously.

